# PET/CT in Prostate Cancer

**DOI:** 10.3390/cancers15153751

**Published:** 2023-07-25

**Authors:** Laura Evangelista, Stefano Fanti

**Affiliations:** 1 Department of Biomedical Sciences, Humanitas University, Via Rita Levi Montalcini 4, 20072 Pieve Emanuele, Italy; 2 IRCCS Humanitas Research Hospital, Via Manzoni 56, 20089 Rozzano, Italy; 3 Division of Nuclear Medicine, IRCCS Azienda Ospedaliero-Universitaria di Bologna, 40138 Bologna, Italy; stefano.fanti@aosp.bo.it; 4 Department of Medical and Surgical Sciences (DIMEC), Alma Mater Studiorum University of Bologna, 40126 Bologna, Italy

Over the last decade, PET/CT has played a crucial role in managing patients with prostate cancer (PCa), significantly impacting various aspects of the disease. A search on PubMed using MESH terms “Positron Emission Tomography” and “Prostate Cancer” yielded over 3100 papers, with the highest number published in 2020 (n = 513 articles). The introduction of new agents like 11C/18F-Choline, 18F-Fluciclovine, and 68Ga/18F-PSMA has posed a significant challenge, but also holds promise, not only for diagnostics but also for therapeutic applications. Radiolabeled PSMA PET/CT has gained substantial interest, as alternative indications have not been fully explored using 11C/18F-Choline and 18F-Fluciclovine. The role of radiopharmaceutical agents in the initial diagnosis of PCa has become a crucial topic. Additionally, the integration of artificial intelligence has emerged as an interesting avenue for improvement in this field.

This special issue includes 14 papers discussing the aforementioned topics, and they are summarized in Table 1, of these papers, ten are original articles, and the remaining four are expert and systematic reviews. The topics covered range from machine learning applications [1,2,3,4] to the detection rate and diagnostic performance of PET/CT imaging [5,6,7,8] and its prognostic value [8,9,10,11,12]. There are also diverse topics covered in the papers [13,14]. Radiolabeled PSMA was commonly used in several papers [1,2,3,5,13,14], along with radiolabeled choline in three papers [4,8,9], and 18F-Fluciclovine in one study [6]. Additionally, two papers employed dual-tracer PET imaging (18F-FDG and radiolabeled PSMA) as a biomarker for predicting a theragnostic approach with 177Lu-PSMA [11,12]. Another paper focused on the treatment of metastatic castrate-resistant PCa (crPCa) with 223Ra [10].

The key take-home messages from these manuscripts are as follows:

1. Radiomic analysis enhances prostate lesion detection and tumor grading prediction, especially for intermediate ISUP-grade lesions or in cases with an undefined PIRADS-3 score. It can also aid in improving the detection of liver metastases in crPCa, as radiolabeled PSMA, mainly 18F-PSMA, may have limitations in physiological biodistribution.
2. The findings of 68Ga-PSMA PET/CT can predict progression-free survival (PFS) in oligometastatic patients treated with metastatic-directed therapy (MDT). Dual-imaging PET with PSMA and FDG before 177Lu-PSMA-617 treatment appears significant, but the meaning of the imaging mismatch should be further evaluated.
3. In the oligometastatic setting, PSMA PET should be carefully interpreted and considered in clinical trials, as about 20% of patients only exhibit PSMA uptake in the bone, and half of them may benefit from MDT.
4. There are unresolved issues including the role of PSMA PET in the initial diagnosis compared to mpMRI, and the utility of PET parameters for evaluating treatment response.

In conclusion, this Special Issue presents valuable research that can enhance the management and prognosis of PCa patients, with the potential to have a significant impact on their outcomes.

## Figures and Tables

**Table 1:** Summary of the published papers in this Special Issue (according to the website: https://www.mdpi.com/journal/cancers/special_issues/PET_CT_Prostate_Cancer, accessed on 17 July 2023).

Authors, Ref.	Type of Article	Number of Patients	Number of Papers	Topic	Setting of Disease (Agent)
Gaudiano et al. [1]	Original article	133	-	Machine learning	Diagnosis (PSMA)
Serani et al. [5]	Original article	179	-	Detection rate	Initial staging and recurrent disease (PSMA)
Vetrone et al. [13]	Original article	44	-	Semiquantitative PET data	Staging (PSMA)
Lanfranchi et al. [9]	Original article	37	-	Prognostic value	Recurrent disease OMD (PSMA and Choline)
Mattoni et al. [2]	Original article	60	-	Radiomics analysis	Initial staging and recurrent disease (PSMA)
Feliciani et al. [3]	Original article	28	-	Radiomics analysis	Diagnosis (PSMA)
Bauckneht et al. [10]	Original article	494	-	Prognostic value	Metastatic crPCa (223Ra)
Nappi et al. [6]	Original article	58	-	Detection rate and performance	Recurrent disease (18F-Fluciclovine)
Harthrampf et al. [11]	Original article	32	-	Prognostic value	Metastatic crPCa (PSMA + FDG)
Khreish et al. [12]	Original article	29	-	Prognostic value	Metastatic crPCa (PSMA + FDG)
Sabbagh et al. [14]	Expert Review	-	NA	OMD	PSMA
Caracciolo et al. [7]	Systematic Review	-	8	Diagnostic performance	Diagnosis (PSMA)
Alongi et al. [8]	Systematic review	-	40	Diagnostic performance and prognostic value	Assessment of response to therapy (Choline and PSMA)
Guglielmo et al. [4]	Systematic review	-	6	Radiomics analysis	Staging (PSMA and Choline)
